# Formula for Precipitated Chalk

**Published:** 1844-07-27

**Authors:** 


### FORMULA FOR PRECIPITATED CHALK.

The Editor of the Pharmaceutical Journal observes, that the extensive use which "precipitated chalk" has recently acquired, as a substitute for the impure "prepared chalk," and the high price which, until lately, has been charged for the former as compared with the latter, have led to several inquiries from correspondents, as to the best and most economical method of making a good "Creta Precipitata." The process that he recommends is this:—

Take of White Marble 1 part.
Pure Hydrochloric Acid, 2¼ parts.
Carbonate of Soda (crystals,) 3 parts.
Water, a sufficient quantity.

Dissolve the marble in the hydrochloric acid, previously mixed with two parts of water, and dilute the solution with four parts more of water. Dissolve the carbonate of soda in twelve parts of water. Mix the solutions, and collect, wash, and dry the precipitate.
*Pharm. Journal.*

